# Are peri-urban land transactions a disaster in the making? A case of Domboshava, Zimbabwe

**DOI:** 10.4102/jamba.v11i3.708

**Published:** 2019-07-03

**Authors:** Emaculate Ingwani

**Affiliations:** 1 Department of Urban and Regional Planning, University of Venda, Thohoyandou, South Africa

**Keywords:** Customary Land Tenure, Land Transactions, Peri-Urban, Risks, Hazards, Disaster

## Abstract

Peri-urban communal areas close to bourgeoning cities in sub-Saharan Africa are increasingly under various disaster threats, from social, environmental and institutional perspectives, as a result of urbanisation and migration. Residents of these communal areas have taken land matters into their hands, which leads to diverse land transactions. This study aimed to emphasise risks and hazards arising from land transactions taking place in a peri-urban zone of Domboshava, Zimbabwe, situated close to Harare, the capital city. Land transactions in this area include land exchanges through buying, renting and, in some cases, land grabbing. Because land transactions are on the increase in Domboshava, risks and hazards could potentially develop in social, environmental and institutional terms. Appropriate planning techniques and principles are solely needed to avoid potential disasters.

## Introduction

Peri-urban communal areas close to bourgeoning cities in sub-Saharan Africa are increasingly under various disaster threats, from social, environmental and institutional perspectives, as a result of urbanisation and migration. Residents of peri-urban communal areas have taken land matters into their hands, which leads to diverse land transactions. This study aimed to emphasise risks and hazards arising from land transactions taking place in a peri-urban zone of Domboshava, Zimbabwe, which is situated close to Harare (the capital city). Land transactions in this communal area include land exchanges through buying, renting and, in some cases, land grabbing. Because land transactions are on the increase in Domboshava, risks and hazards could potentially develop in social, environmental and institutional terms. The findings remain imperative in generating responsive policy initiatives in disaster risk reduction portfolios for peri-urban zones.

### Background to Domboshava

The peri-urban communal area of Domboshava is situated 20 km northeast of Harare in Ward 4 of Goromonzi District, Mashonal and East Province, Zimbabwe. This area is emerging as a development node for surrounding communal areas and farms in Goromonzi District owing to its proximity to Harare, the capital city. Domboshava is rapidly accumulating urban population from Harare, as well as people from other places across the country, as a result of incessant land transactions between residents of local tribal descent and migrants. Tribal members hold historically sanctioned communal land rights under the system of customary land tenure, whereas migrants do not have legitimate lineage land rights in the communal area because they migrated from elsewhere to live in this communal area. As more migrants settle in Domboshava, diverse, unique and complex experiences are generated in this peri-urban zone.

Domboshava is a rural area under traditional authority and a local authority called Goromonzi Rural District Council. Land in Domboshava falls under communal land tenure system and is administered under the system of customary land tenure. Statutes on land and settlement, on the one hand, and those on local customs and tradition, on the other hand, legally constitute a structure that regulates access to land in this peri-urban communal area. The aim of this study was to emphasise the risks and hazards arising from land transactions in the peri-urban communal area of Domboshava. From a spatial and regional planning perspective, land transactions interfere with land use activities in rural areas – mainly residential, arable and grazing. As land transactions are on the increase, these activities now take place outside the obligatory land use management schemes. This is likely to generate risks and hazards that could turn into social, environmental and institutional disasters.

### Conceptualising land transactions and disasters

Land transactions in Domboshava involve land exchanges within customary land tenure (customary system) or outside of it (non-customary or individualised system). Customary land transactions involve inheritance of land through the tribal line, while individualised land transactions involve direct land sales, renting and land grabs. Table 1 shows the customary and individualised land transactions (2002–2012) recorded during a fieldwork in Domboshava.

**TABLE 1:** Categories of land transactions recorded in Domboshava.

Categories of land transactions		Before 2002	Between 2002 and 2012	Total
**Customary land transactions**				
**Inheritance**	With a homestead	5	2	7
	Without a homestead	12	5	17
**Individualised land transactions**				
**Direct land sales**	With a homestead	1	0	1
	Without a homestead	6	28	34
**Renting**	With a homestead	2	7	9
	Without a homestead	0	2	2
**Land grabs**	With a homestead	0	0	0
	Without a homestead	3	4	7

**Total**		29	48	77

*Source*: Hungwe (2014:158)

Trends in Table 1 demonstrate the prevalence of individualised over customary land transactions during the 2002–2012 period. As only 41 households participated in this research and 77 land transactions were recorded in total, it shows that some community residents were involved in compound or multiple land transactions. This clearly demonstrates looming disaster(s) from unsanctioned land exchanges and increased settlement densities as more people settle in the peri-urban communal area of Domboshava.

**FIGURE 1:**
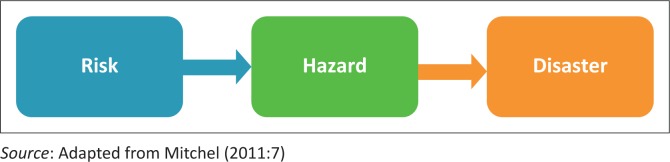
A depiction of progression from risks to disasters.

In conceptualising risks, hazards and a disaster ‘in the making’, reference is made to a simple progression of these phenomena postulated by Mitchel (2011) – see Figure 1. A disaster entails a disruption of the expected functioning of a community or a society, often involving losses and negative impacts (International Strategy for Disaster Reduction 2009; Mitchel 2011). Disasters present crises situations that incapacitate affected individuals to cope with loss of assets and services (Mitchel 2011). Often, disasters originate from prevailing risks, which turn into hazards if not managed. On the other hand, risks relate to exposure to negative consequences embedded in social systems (United States Agency for Development (USAID 2011). Hazards represent harmful phenomena or human activity that lead to undesirable conditions, especially change in livelihood strategies (USAID 2011). Clearly, risks and hazards can turn into disasters if left unchecked. Risks, hazards and disasters can be natural or anthropogenic and of varied intensity. It is therefore important to analyse the context in which risks and hazards are presented in social systems, such as the system of customary land tenure, in order to understand the emergence of a possible disaster ‘in the making’. This study highlights the different kinds of risks and hazards arising from land transactions (customary or individualised form) in the peri-urban communal area of Domboshava.

In analysing data, reference is made to the structure–agency debate postulated by Antony Giddens (1999). The basic elements of Giddens’ structuration theory are the structure and agency. The system of customary land tenure is the structure, while individual action forms agency; these are inseparable. From this viewpoint, people who live in communal areas are perceived to possess ability to change the structure around them to their advantage through social interaction. The outcomes from these individual actions can be positive or negative, which could generate risks and hazards that can potentially develop into disasters, if not managed.

## Methods

For this study, a mixed methods approach (a combination of qualitative and quantitative approaches) was adopted from Creswell and Clark (2011). However, priority was given to the qualitative approach because the main focus of the research was to characterise a disaster ‘in the making’ as community residents exchange land and seek survival in the peri-urban communal area of Domboshava. The research also adopted a case study design. Domboshava peri-urban communal area was purposively selected for this research because it provides a unique case on land transactions and the likely peri-urban disasters. The study focused on four villages, with a sample of 41 households, as well as a number of key informants such as traditional leaders and local government officials. Qualitative data were collected through structured interviews, as well as observation during fieldwork. Data were also collected through review of pertinent documents.

### Identifying risks from land transactions

The risks and hazards evident in Domboshava owing to the prevalence of land transactions and the peri-urban nature of this communal area are highlighted. This section demonstrates how this situation can potentially develop into a disaster. Community residents (of both tribal and migrant descent) are capable of breaking rules that regulate land transactions through agency. In the process, they produce both intended and unintended consequences. These outcomes are capable of generating disaster risks and hazards in social, economic, environmental and institutional terms as individuals interact with the structure for survival.

#### Inheritance of tribal land in Domboshava by migrants

While inheritance is regarded a common practice among tribal households, migrants that acquire land rights also assume the right not only to pass their land rights to migrant descendants through inheritance despite the individualised motive behind their land transactions, but also to sell or rent such land. In the minds of migrant households, migrant descendants have the right to inherit communal land in Domboshava. Yet, under the system of customary land tenure, migrants can only assume use rights. These circumstances present a source of conflict as land transactions within the system of customary land tenure remain a prerogative of traditional leaders and tribal members. Not only is there a conflict between the traditional leaders and the statutes on land and settlement but also an institutional conflict concerning migrants’ inheritance of tribal land. Migrants assume that they could in traditional terms (and not in legal terms) pass on land as inheritance to their descendants through customary land transactions. Although inheritance poses conflict and risks in terms of shifts in the structure that defines customary land exchanges, it remains an important institution that ensures passage of land rights to household members, as well as securing these rights at the same time.

#### Migrants distribute communal land to fellow migrants

Tribal members lose their prerogative in land allocation in Domboshava as migrants often sell land to other land seekers. This demonstrates that tribal authority is slowly diminishing as migrants not only assume land rights but also assume, in some instances, tribal roles in land allocation to fellow migrants and to some tribal members. This marks the departure from traditional values and statutory procedures on land allocation under the system of customary land tenure, as this role is a prerogative of traditional leaders and tribal heads of households. This situation not only creates new land tenure regimes but also reproduces new forms of securing the acquired land rights within the current system of customary land tenure.

#### Reduction of arable land supposedly meant for peasant farming

When the community residents of Domboshava engaged in land transactions, their choices seemed simple, but the outcomes were variable and complex through significant reduction in arable spaces. Community residents need to survive through land transactions while they secure their land rights at the same time. They did not intend to destabilise the status quo, but were simply responding to surrounding circumstances. This creates shifts in household survival strategies from farm to off-farm activities. For example, during the 1960s, tribal households of Domboshava were allocated three acres of fields, one acre of a garden and one acre of the yard – by the then Land Development Officers. Fifty years later, none of the surveyed households in Domboshava owns these original land parcels in full. Yet, tribal members simply expanded households’ income streams through land transactions, catered for new household formations and extended assistance to victims of displacement from urban areas and adjacent farms. As a way of coping to reduce arable land, some tribal members resort to sharing their portions of vlei gardens and arable spaces.

#### Loss of tribal legitimacy and authority

As tribal members practised individualised land transactions such as direct land sales to migrants, they inversely transformed their territorial boundaries in both physical and institutional terms. Sold land translates to reduced land holding capacities of individual tribal households, as well as tribal land collectively. As more and more migrants settle in Domboshava, they occupy more territorial space than tribal members. Thus, individual action and choices to practise direct land sales engender collective consequences in terms of loss of territorial space and community identity. Identities are constructed through occupying specific territorial spaces (Gervais-Lambony 2006). In institutional and physical terms, land is an integral property and economic resource that serves in the production of wealth, as well as a territory in terms of a governed space that gives those land controllers leverage to control others (Berry 2008:27 in Peters 2010:604).

Reduction of tribal land through individualised land transactions presents new sets of conflict and later on, risks in terms of loss of legitimacy of traditional leaders as the custodians of tribal authority and land under the system of customary land tenure. The chief is increasingly losing his legitimacy in Domboshava. Legitimacy of Chiefs can be defined through dominance of tribal members often of the same lineage within a communal area and the breadth of the territories they govern (see Andersson 1999). Land in communal areas defines not only the existence of communities under traditional leaders such as chiefs but also the existence of territorial space under their command. Rural communities in Zimbabwe are not only geographic entities characterised by people who occupy spaces and boundaries but also cohesion of these people as they share common property resources at their disposal under the custody of chiefs (Andersson 1999; O’Flaherty 1998). Part of the risks visible in Domboshava because of individualised land transactions (especially, direct land sales and land grabs) is therefore the disappearance of the tribal community in spatial, territorial and institutional terms – something both tribal and migrants did not intend in the first place. Migrants emerge as winners from land transactions as they access additional land rights apart from their autochthons rights in their homelands, whereas tribal members lose their tribal land rights, territory and legitimacy to migrants.

### The identified hazards from land transactions

If risks are unmanaged, they culminate into hazards, thereby increasing the vulnerability and inability of communities to cope with undesirable situations. As such, hazards emerge as a result of unmanaged impacts of risks on society. This section posits the likely hazards that emerge from the conduct of community residents of Domboshava as they interacted with the structure that defines land transactions under the system of customary land tenure.

#### Land use change and disappearance of the commons

Both customary and individualised land transactions in Domboshava result in change of land use categorised as arable, grazing, residential and the commons. Arable land constitutes the fields and vlei gardens, whereas the commons comprise forests, grazing lands, wetlands and watersheds. As a result of land transactions such as inheritance and land sales, the commons and arable land reserved for peasant farming are slowly degenerating into residential spaces. Yet, peasant farming generally forms the backbone of livelihoods in most communal areas of Zimbabwe. The traditional hallmark for survival of most tribal households of Domboshava – the vlei gardens – is turning into residential spaces. As a result, Domboshava is losing its title as the ‘Tomato Kingdom’ [*Kumadomasi*] as most tribal households now depend on peasant producers of vegetables in other villages.

**FIGURE 2:**
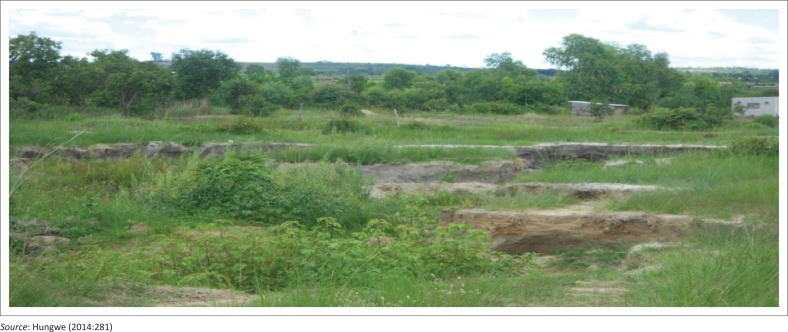
Degradation of the commons as a result of increased land transactions in Zimbiru village of Domboshava.

In addition, the natural extinction of natural habitats for a variety of animal and plant species is evident as vlei gardens and common property resources such as grazing, forests, wetland ecosystems and watersheds degenerate into residential spaces. Degraded spaces are also rendered unusable for peasant farming owing to extraction of sand and quarry for construction purposes. Figure 2 shows the extent of land degradation as a result of land transactions in some parts of Zimbiru village.

According to the International Strategy for Disaster Reduction (2009), environmental degradation as a hazard reduces the capacity of the environment to satisfy its ecological and social obligations. Land degradation induces community vulnerability on livelihoods because land is a community asset for peasant farming and grazing.

#### Change in settlement pattern and settlement density

The settlement pattern and settlement density in Domboshava are altered because of an increased number of migrants settling in this communal area. The layout of the residential spaces in Domboshava changed greatly from a typical rural settlement (where homesteads are scattered) to more closely settled homesteads (which are overcrowded and messy). In Domboshava, new building structures of modern outlook are juxtaposed, with traditional structures often dilapidated. This creates a mixed settlement ‘far from being rural’. For example, new homesteads depict a modern outlook compared to some traditional residential structures. Migrants who buy large residential spaces construct up-market houses in most cases with a roof under tiles, gated and with pre-cast walls. In most cases, dwelling units for tribal members have a traditional outlook such as roofs under thatch, traditional rounded kitchens separated from the main houses and fowl runs. Differences between physical structures of homesteads account for differential outcomes from land transactions mostly unintended in terms of rich migrants or poor tribals and rich tribals or poor migrants. In order to cope with these social hazards and inequalities, some tribal members invest in building modern structures, while some tribal children build homesteads for their parents as a way of improving their social conditions, and disrupting distortions and class distinctions that emanate from mixed settlements.

In any case, land use planners and Local Government Officers are greatly concerned that Domboshava is degenerating into an informal settlement such as Epworth situated in the north-eastern part of Harare. Land transactions result in unplanned settlement and urban sprawl. This presents planning risks that are capable of generating hazards and disasters usually associated with informal settlements and slums. Unplanned urbanisation exposes community residents to hazards of many kinds (Mitchel 2011).

#### Increase in population densities and overcrowding

**FIGURE 3:**
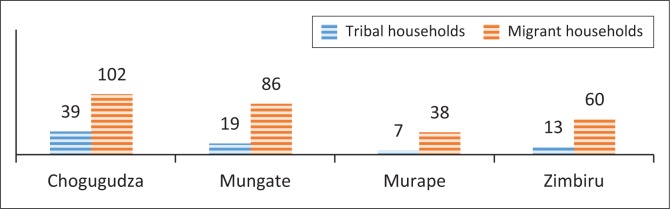
The number of households in each of the four surveyed villages.

Migration is one of the major contributory factors to changes in demographic patterns of Domboshava. The trends in population increase in Domboshava signify future scenarios on domination of land rights by migrant households compared to tribals, as well as the likely shifts in customary land rights in this communal area. Because of population increase in Domboshava, an increase in social hazards of crime such as ritual murders, muggings, stock theft and crop theft has been reported (Share 2012). Figure 3 shows the number of households recorded in each village.

Clearly, migrant households constitute the bulk (79%) of the households in the four villages studied. These findings also signal possible hazards in terms of future demands for land rights under customary land tenure as children grow old, marry and establish their homesteads in Domboshava. As the population of Domboshava increases, households’ land parcels would be divided as their members establish themselves through marriage. Population pressure often undermines the ability of rural residents to generate income through agriculture (Delius & Schirmer 2001). This in turn increases the vulnerability of community residents to food insecurity. Although the community residents can adapt to population increases, they have limited ability to cope with risks and hazards arising from land transactions, such as loss of arable land to practice peasant farming.

#### Looming health hazards from unplanned settlement

Observations reveal that most water wells and ‘Blair’ toilets are haphazardly sited in proximity to each other, particularly in Zimbiru Village. Under these circumstances, water from wells is at risk of contamination as revealed by the vignette in Box 1. Poor siting of toilet facilities and water wells exposes people to unhygienic health hazards such as water-borne diseases, as highlighted in Box 1 by one of the heads of households from Domboshava.

BOX 1 Views on the possibility of water contamination in Domboshava.

‘People are so crowded especially those close to the shops. In terms of disease outbreak, it can be a disaster. It is a health time bomb. These people use open wells. The borehole was only repaired recently. It is very unfortunate that those who buy land have money, and they are able to build beautiful and standard houses with toilets. Some homesteads do not have toilets. Some use pit latrines that collapse especially during this rainy season. If you take a look at those homesteads, they do not have toilets (pointing). We were surprised that the school fence was cut each time it was repaired. People from those homesteads use a hole in the fence to gain entry into the schoolyard to access the school toilets. Now, the school gate is left open to allow people to use the school toilets. The NGOs should provide toilets like what is happening in other communal areas. The RDC’s intervention should prioritise toilets at each homestead’.

*Source*: Hungwe (2014:198)

### Ethical consideration

Research ethics were observed during the data collection process.

## Conclusion

The prevalence of land transactions in the peri-urban communal area of Domboshava presents complex patterns of intended and unintended outcomes as community residents interact with the structure that regulates land transactions under the customary land tenure system. The outcomes are slowly progressing from risks to hazards. Disasters in social, economic, environmental and institutional terms are ‘in the making’ as highlighted through the findings of this research. These types of disasters are often difficult to manage because they are complex. However, the conflicting and complex outcomes from the interactions of community residents of Domboshava with the system of land tenure appear as inevitable because of the peri-urban nature of this communal area, as well as the presence of continuous change. In any case, peri-urban contexts by nature are characterised by heterogeneity of activities and processes. This situation is likely to prevail until reforms to the administration of land and access to customary land rights in peri-urban zones are applied, and desirable planning techniques are implemented. Appropriate planning techniques are solely needed to avoid a disaster ‘in the making’ in Domboshava.
